# Community‐level phylogenetic diversity does not differ between rare and common lineages across tallgrass prairies in the northern Great Plains

**DOI:** 10.1002/ece3.9453

**Published:** 2022-11-01

**Authors:** Sarah A. Herzog, Maribeth Latvis

**Affiliations:** ^1^ Department of Natural Resource Management South Dakota State University Brookings South Dakota USA; ^2^ C.A. Taylor Herbarium South Dakota State University Brookings South Dakota USA; ^3^ Division of Biology Kansas State University Manhattan Kansas USA

**Keywords:** community assembly, conservation, grassland, niche differentiation, phylogenetic diversity, rare species

## Abstract

Niche differentiation has served as one explanation for species coexistence, and phylogenetic relatedness provides a means to approximate how ecologically similar species are to each other. To explore the contribution of rare species to community phylogenetic diversity, we sampled 21 plant communities across the Prairie Coteau ecoregion, an area of high conservation concern. We used breakpoint analysis through the iterative addition of less abundant species to the phylogenetic tree for each community to assess the contribution of rare species to community phylogenetic diversity. We also quantify the phylogenetic signal of abundance using Blomberg's K statistic and calculated the phylogenetic similarity between rare and common species using a phylogenetic beta‐diversity metric (*D*_nn_). To estimate the phylogenetic structuring of these prairie communities, we calculated two common metrics that capture evolutionary relatedness at different scales (MPD and MNTD). Additionally, we examine the correlation between Faith's PD, MPD, and MNTD and species richness. We found rare species do not generally contribute higher levels of phylogenetic diversity than common species. Eight communities had significant breakpoints, with only four communities having an increasing trend for the rarest species. The phylogenetic signal for abundance was low but significant in only four communities, and communities had lower phylogenetic diversity than expected from the regional species pool. Finally, the strength of the correlation between species richness and phylogenetic diversity was mixed. Our results indicate niche differentiation does not explain the persistence of rare species in tallgrass prairies, as they were more closely related than expected from random, suggesting high functional redundancy between rare and common species. This is promising for the long‐term resilience of this ecosystem, but only insofar as enough species remain in the system. With ongoing biodiversity loss, it is essential that we understand the role rare species play in their communities.

## Graphical abstract

Niche differentiation theory predicts rare species are maintained in communities through niche differentiation. Using phylogenetic diversity, we find plant communities in tallgrass prairies of the Great Plains are closely related, with rare species being closely related to more common species and other rare species, rejecting this hypothesis.

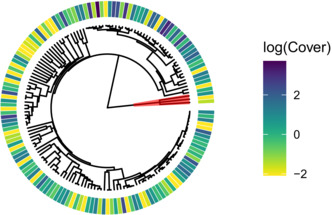

## 1 INTRODUCTION

Biodiversity loss has accelerated over the past century (Barnosky et al., 2011; Ceballos et al., 2015), with 75% of terrestrial ecosystems having been significantly altered by human activity (Venter et al., 2016). Among these, temperate grasslands are one the most threatened ecosystems owing to extensive habitat loss and lack of protection (Hoekstra et al., 2005). Patterns of species loss are non‐random and may disproportionately impact some lineages more than others for several reasons, including evolutionary relatedness (Davies & Yessoufou, 2013; Purvis et al., 2000), specialization, and population size (reviewed in O'grady et al., 2004). Population size has emerged as the best correlate of extinction risk (O'Grady et al., 2004), with rare species in communities among the most vulnerable (Purvis et al., 2000; Rabinowitz, 1981; Wilfahrt et al., 2021) because of greater susceptibility to environmental and demographic stochasticity (e.g., Lande (1993), Smith and Knapp (2003), and Enquist et al. (2019)). Additionally, rare species generally have narrower niches, more conservative resource acquisition, and/or slower growth rates relative to their more common neighbors (Farnsworth, 2007; Grime, 1977; Rabinowitz, 1981; Rabinowitz et al., 1984). As most species are rare (Brown, 1984; Preston, 1948), it is imperative that we understand how they influence community structure and the potential impacts of their loss to better inform management strategies to ensure their long‐term survival.

The maintenance of rare species in a community has been a long‐standing debate in ecology (Hanski, 1982; Preston, 1948; Soule, 1986). One possible explanation is niche differentiation, where rare species utilize resources less used by common species (Gaston, 1994; Grime, 1998; Hanski, 1982). Niche differentiation theory predicts rare species will be dissimilar in form and function to common species and other rare species, versus a neutral model where there are no significant differences between species (e.g., Hubbell (2005)). Previous work has used evolutionary relationships as a proxy for ecological similarity with the assumption that if rare species are distantly related to abundant species within a community (i.e., phylogenetic overdispersion), they are more likely to exhibit novel traits (e.g., Cadotte et al., 2008; Webb et al., 2002; Wiens et al., 2010). Conversely, if rare species are closely related to common species (i.e., phylogenetic clustering), they may share many of the functional traits of those common species. This creates functional redundancy in the community, which may dampen the effects of diversity loss (Diaz & Cabido, 2001; Walker et al., 1999; Yachi & Loreau, 1999). Additionally, if rare species increase in abundance following disturbance, replacing more common species, they are likely to become even more important in preserving ecosystem functionality (MacDougall et al., 2013).

Initially proposed as a proxy for functional diversity, phylogenetic diversity (Faith, 1992) is a biodiversity metric that aims to quantify the evolutionary history contained within a community by measuring the phylogenetic *branch lengths* that connect all species within that community. Phylogenetic structure (e.g., mean pairwise distance and mean nearest taxon distance) quantifies the *branching patterns* of community phylogenies (Webb et al., 2002). Confusingly, “phylogenetic diversity” may also broadly serve as an umbrella term for several metrics that incorporate evolutionary relatedness to quantify biodiversity, including those that describe phylogenetic structure. Here, we use “phylogenetic diversity” in the broad sense and specify Faith's PD (PD_Faith_) to denote the metric defined by the sum of the branch lengths connecting species in a community (Faith, 1992). The idea that phylogenetic relationships serve as a proxy for ecological similarity has provided a foundation to test numerous hypotheses of community assembly (e.g., Marx et al. (2017), Peterson et al. (2021)), invasion biology (e.g., Marx et al. (2016), Qian and Sandel (2017)), etc. However, this assumption has been critiqued (see Gerhold et al. (2015)), as there may be little phylogenetic signal in commonly measured traits that provide insights into an ecological function (Swenson et al., 2012b; Swenson, Erickson, et al., 2012). While phylogenetic diversity and functional diversity yield different yet complementary information (Hipp et al., 2015; Jones et al., 2019), they are often highly positively correlated (Cadotte et al., 2019). Phylogenetic information provides a good initial look at community structure, particularly when trait data are absent and/or sampling is an obstacle, such as in species‐rich assemblages or for rare species (Swenson, 2013). Thus, various phylogenetic diversity metrics can be used to determine the degree of relatedness between common and rare species, providing a means to evaluate the niche differentiation hypothesis for species lacking trait data. Previous work on the phylogenetic distinctiveness of rare species has indicated mixed support for the niche differentiation hypothesis, with rare species evolutionarily distinct from common species in some communities (Anderson et al., 2004; Kelly et al., 2008; Mi et al., 2012), lending support for the hypothesis, while rejecting it in communities dominated by disturbance (Mi et al., 2012).

Beyond identifying the potential roles that rare species play in a community, metrics that incorporate evolutionary history can be more broadly applied to conservation, as increasing phylogenetic diversity has been shown to lead to more productive communities (Cadotte et al., 2008), increase ecosystem stability (Cadotte et al., 2012), and increase resistance to invasion (Davies et al., 2011; Li et al., 2015). Phylogenetic diversity also appears to capture more variation in biomass than taxonomic richness alone (Cardinale et al., 2012). While phylogenetic diversity is gaining traction as an informative metric for conservation prioritization, the majority of management plans rely on species richness for establishing priority areas due to its ease of calculation and comparison across communities (Howard et al., 2020; Meir et al., 2004; Myers et al., 2000). However, in some cases, species richness does not correspond to the most evolutionarily diverse communities (e.g., Forest et al. (2007), Daru et al. (2019), Brum et al. (2017), Pollock et al. (2017)), yet in other communities it does (Pérez‐Losada et al., 2002; Rodrigues & Gaston, 2002). This may indicate that the relationship between phylogenetic diversity and species richness may need to be examined case by case for communities to understand community assemblage patterns and its impacts on conservation prioritization and management.

Focusing on critically threatened tallgrass prairie communities in the Prairie Coteau ecoregion (northern Great Plains, USA), our first objective is to investigate the contribution of rare species, here defined by rank abundance, to PD_Faith_, which will allow us to evaluate the niche differentiation hypothesis across 21 grassland plant communities. Our second objective assesses the phylogenetic structure of our sampled communities as a whole, and between rare and common species within those communities by using two phylogenetic structure metrics, mean pairwise distance (MPD), and mean nearest taxon distance (MNTD). Niche differentiation theory posits that rare species are maintained within a community because they are evolutionarily/ecologically distinct from common species and each other (Gaston, 1994; Kunin & Gaston, 1993) and significantly increase community phylogenetic diversity. However, because grassland communities tend to be dominated by only a few angiosperm families (Towne, 2002), we predict species within our communities would be more closely related to each other than by chance. This phylogenetic clustering could then result in rare species being closely related to other species in the community, refuting the niche differentiation hypothesis. Our third objective examines the relationship among PD_Faith_, structure metrics (MPD, MNTD), and species richness to broadly assess how species richness impacts relatedness among community members. Finally, we examine the effects of grazing on our selected phylogenetic metrics, which may influence community structure (Hickman et al., 2004; Salgado‐Luarte et al., 2019). Tallgrass prairies have been reduced to <1% of their historical range due to conversion to row‐crop agriculture and grazing (Lark et al., 2015; The Nature Conservancy, 2010; Wright & Wimberly, 2013), and understanding community composition and the role of rare species in these communities will inform which taxa, evolutionary lineages, or traits are most at risk or resilient (Hipp et al., 2015; Jones et al., 2019).

## 2 METHODS

### 2.1 Sites

**FIGURE 1:**
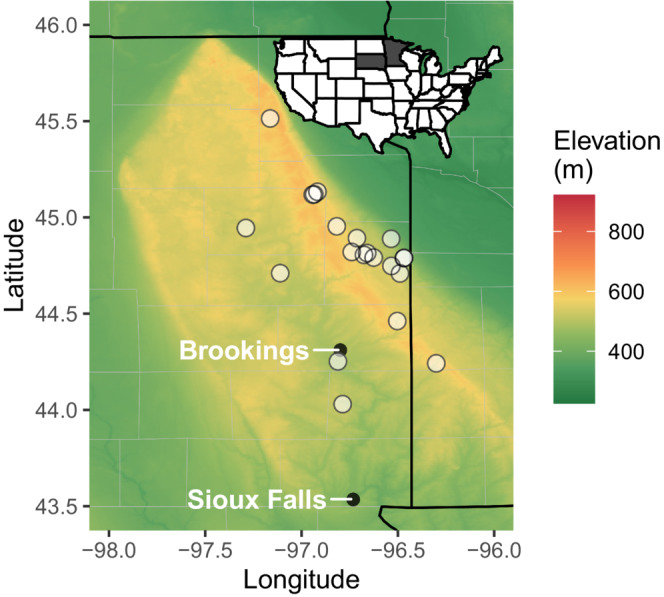
Topographic map of data collection sites (white points). Local cities of Brookings and Sioux Falls are noted

The Prairie Coteau is a Wisconsin‐age glacial moraine, extending from just north of the North Dakota to South Dakota border in Sargent County, North Dakota, through 17 counties in South Dakota and 11 counties in Minnesota. The elevation of the Prairie Coteau ranges from 381 to over 610 m above sea level. The high concentration of protected land in the Prairie Coteau allows for the selection of tallgrass prairies in a range of ownerships and management strategies including communities dominated by invasive non‐natives, untilled remnants, seeded, grazed, and burned sites; however, in many cases, detailed management history is unknown, which prohibited replication across management type (Table 1). Communities were selected based on the expertise of South Dakota Game Fish and Parks, U.S. Fish and Wildlife Services, The Nature Conservancy (TNC), and South Dakota State University (SDState) extension personnel and ranged in size from 20 to 1674 acres. Landowners included South Dakota Game Fish and Parks, U.S. Fish and Wildlife Services, TNC, and the City of Brookings, SD. In total, 21 communities were utilized across 19 sites (two localities were large and had varying management practices employed and were treated as separate communities; Figure 1 and Table 1).

**TABLE 1:** List and locations of sampled communities

Community	Latitude	Longitude	State	County	Elevation (m)	Date visited	Owner	Grazed
7‐mile	44.74872	−96.53342	SD	Deuel	504	7‐Aug‐19	TNC	no
Altamont Prairie	44.88957	−96.53481	SD	Deuel	443	9‐Aug‐19	TNC	yes
Brookings Prairie	44.2533	−96.81075	SD	Brookings	483	29‐Aug‐19	City of Brookings	No
Coteau Lake	44.82095	−96.73891	SD	Deuel	563	11‐Aug‐19	State	
Coteau Prairie	44.89298	−96.7121	SD	Deuel	545	13‐Aug‐19	Federal	
Cox	44.71103	−97.11017	SD	Hamlin	535	21‐Aug‐19	Federal	
Crystal Springs	44.81537	−96.65897	SD	Deuel	574	9‐Aug‐19	State	Yes
Deer Creek	44.46254	−96.503	SD	Brookings	535	27‐Aug‐19	State	Yes
Gary Gulch E	44.79012	−96.46774	SD	Deuel	474	14‐Aug‐19	State	No
Gary Gulch W	44.78933	−96.46859	SD	Deuel	474	8‐Aug‐19	State	Yes
Hole in the Mountain	44.24301	−96.30058	MN	Lincoln	565	22‐Aug‐19	TNC	
Jacobson Fen	44.79181	−96.62524	SD	Deuel	529	7‐Aug‐19	TNC	No
Lake Ketchum	44.80579	−96.67554	SD	Deuel	538	12‐Aug‐19	State	No
McKillican	44.94546	−97.28811	SD	Codington	535	15‐Aug‐19	State	
Overland	45.13369	−96.9169	SD	Codington	580	19‐Aug‐19	Federal	
Punished Woman Grazed	45.11885	−96.94109	SD	Codington	575	16‐Aug‐19	State	Yes
Punished Woman Seeded	45.12052	−96.93369	SD	Codington	569	19‐Aug‐19	State	No
Round/Bullhead	44.9542	−96.8169	SD	Deuel	575	13‐Aug‐19	State	No
Severson	44.70961	−96.48895	SD	Deuel	525	6‐Aug‐19	Federal	Yes
Sioux Prairie	44.0313	−96.78635	SD	Moody	517	23‐Aug‐19	TNC	No
Wike	45.51416	−97.16224	SD	Roberts	599	20‐Aug‐19	Federal	No

### 2.2 Sampling design

Communities were visited once between August 6 and August 29, 2019. Transect sampling methods were based on Barak et al. (2017): two 50 m transects were randomly placed at each site. A random number generator was used to dictate the direction of the transect and number of steps from the entry point. 0.25 m^2^ square quadrats were placed every 5 m, resulting in 10 quadrats placed for each transect and 20 quadrats for each community. Quadrats were randomly placed 2 to 7 m (1 m increments) away from the main transect line on the left or right. Abundances for each species were visually estimated using the Daubenmire six cover class method and using the midpoint percent cover values (Daubenmire, 1959).

All individuals found in a plot were identified in the field. For those species in reproductive condition (i.e., with flowers, fruits, or producing spores) and with multiple individuals present in the immediate area, we additionally collected vouchered herbarium specimens in triplicate to aid in the identification and contribute to long‐term biodiversity monitoring efforts. Species were identified using *The Vascular Plants of South Dakota* (Van Bruggen, 1985) with verification from taxonomic experts. All vouchers were digitized and are curated by the C.A. Taylor Herbarium (SDC) at SDState with records available on the Consortium of Northern Great Plains Herbaria (http://ngpherbaria.org/). Additionally, leaf tissue was preserved in silica gel to create a DNA repository from all vouchers and deposited at SDC.

### 2.3 Regional phylogeny estimation

Using all species from across all plots, we created a regional phylogeny using the R (R Core Team, 2018) package “V.PhyloMaker” version 0.1.0 (Jin & Qian, 2019), which constructs a synthesis phylogeny from a species list based on a dated mega‐tree derived from Zanne et al. (2014) and Smith and Brown (2018), and includes 74,533 species covering all extant vascular plant families. Species names were updated using the Catalogue of Life checklist (Bánki et al., 2022) prior to assembling the tree. For the purposes of calculating phylogenetic diversity metrics, our regional phylogeny consisted of only species observed at our sites rather than a comprehensive checklist for the Northern Great Plains. The resulting synthesis phylogeny is scaled to time and contains molecular branch lengths. Phylogenetic diversity values calculated from synthesis phylogenies (such as the “V.PhyloMaker” approach) strongly correlate with those calculated from phylogenies constructed directly from gene sequence data (Allen et al., 2019; Jantzen et al., 2019; Li et al., 2019b).

Taxa within our communities not present in the mega‐tree were grafted to the resulting synthesis tree as follows: missing species belonging to a genus already present in the synthesis phylogeny were bound to the basal node of the genus. For species identified only to the genus level, we used the *add.species.to.genus* function in the “phytools” package (version 1.0–3) in R (Revell, 2012) to add a “[*Genus*] *sp*.” branch from the most recent common ancestor node for the genus. One collection in Cyperaceae was not identifiable to the genus and was removed in subsequent analyses. While *Equisetum* (Equisetaceae) was found in seven communities, we focused our downstream analyses to only angiosperm species following Kellar et al. (2015).

### 2.4 Quantifying the contribution of rare species to community phylogenetic diversity

We used Faith's phylogenetic diversity (PD_Faith_), defined as the sum of phylogenetic branch lengths connecting all species in a community, to calculate community diversity (Faith, 1992). Following Mi et al. (2012), species were first ranked by percent cover for each community and PD was calculated in a stepwise fashion by adding increasingly rare species as follows: First, we calculated PD_Faith_ for the two most abundant species, followed by the three most abundant species and so forth. This process was iterated until the rarest species had been added to the tree and PD_Faith_ was calculated. Due to multiple species sharing the same percent cover values, these calculations were run for 1000 iterations and the mean value for each species rank was used for later analyses. To standardize metrics across communities, we calculated standardized effect sizes (SES) for PD_Faith_: SES = (PD_observed_ – mean [PD_expected_])/sd(PD_expected_). Null (expected) phylogenies were created using “tip shuffling” and 999 randomizations using R package “picante” (version 1.8.2). This method of randomization shuffles the names of taxa across the phylogenetic tree, resulting in branch length randomization without modification of the distribution or total tree branch lengths. SES were calculated using a custom function that maintained “tip shuffling” randomization based on code from Swenson (2014).

After PD_Faith_ was calculated for each abundance category and its corresponding community, we modeled the relationship between SES of PD_Faith_ (SES_Faith_) and the abundance category to generate a “species rank abundance PD_Faith_” (SAPD) curve (Mi et al., 2012), which depicts PD_Faith_ on the y‐axis and increasingly rare ranks on the x‐axis. If rare species are contributing high levels of phylogenetic diversity to the community, we would expect to see an increase in SES_Faith_ values (slope > 0). If rare species contribute low levels of phylogenetic diversity (are closely related to common species), we would expect a decrease in SES_Faith_ (slope < 0). And finally, if rare species add random amounts of phylogenetic diversity, we would expect the SES_Faith_ values to stay relatively constant (slope = 0) or random fluctuations (Mi et al., 2012). As multiple trends are possible along the SAPD curve, a piecewise regression (breakpoint analysis) was conducted for each community to test for one to three breakpoints. The maximum of three breakpoints was based on visual estimation of the SAPD trendlines. To create a piecewise regression, we started by creating a simple linear model and then looked for the presence of a breakpoint in the linear trend by testing for a non‐zero difference in slope parameters (alpha = 0.05). We used the Bayesian information criterion (BIC) to estimate the number of breakpoints (Schwarz, 1978), with the identified number of breakpoints corresponding to the model with the lowest BIC value. If breakpoints were found close to a boundary (within the first four abundance ranks denoting the most common species), we re‐ran the analysis with one less breakpoint, as this region did not contain any rare species and thus was not of interest. All breakpoint analyses were conducted using the “segmented” package (version 1.5–0) in R (Muggeo, 2003, 2008).

### 2.5 Phylogenetic signal of abundance

To further test the relationship between abundance and evolutionary relatedness, we calculated the phylogenetic signal of abundance, defined by percent cover in each community. We used Blomberg's K statistic (Blomberg et al., 2003), which compares the observed levels of the phylogenetic signal of abundance in our communities to the level of phylogenetic signal under a Brownian motion model. Abundance data were log‐transformed prior to testing to reduce variation in the variance. If *K* > 1, the abundance categories show higher levels of phylogenetic signal than expected from Brownian motion (i.e., species with similar abundances are clustered), versus a *K* < 1 indicating less phylogenetic signal than expected. The significance of the phylogenetic signal was assessed by comparing observed abundance patterns to a null model established by shuffling taxa labels across the phylogenetic tree in 1000 iterations with a null hypothesis of *K* = 1. Traits with a *p‐*value <.05 have non‐random phylogenetic signal. The K statistic was calculated using the “phytools” package in R using average species cover across all quadrats and for each community independently.

### 2.6 Evolutionary relatedness among and between rare and common species

To examine evolutionary relationships *between* rare and common categories, we calculated *D*_nn_, a form of phylogenetic beta diversity (Webb et al., 2008) that measures the SES of the mean nearest taxon distance (MNTD) between common and rare species in a community (SES_D_). To calculate SES_D_, we first created separate categories for rare and common species by ranking the mean cover for each species at each community and calculating the percentile for each species at each community. We used three thresholds to define rarity: 0.25, 0.50, and 0.75, where species ranked lower than these percentiles were considered “rare.” If fewer than four species were in the common or rare group for a community using a certain threshold, that community was removed from analyses for that threshold. Where MNTD is the mean phylogenetic distance between a species and its closest relative for each species across a community, *D*_nn_ is the mean phylogenetic distance between a species and its closest relative *in the other category*. Thus, to explore relatedness *within* rare and common categories, we relied on SES_MNTD_. Positive SES values indicate taxa in the community are more distantly related to each other than would be expected by chance (phylogenetic overdispersion) and negative values indicate taxa are more closely related to each other than random chance (phylogenetic clustering). We used tip shuffling for 999 iterations to calculate SES values and the “picante” package, version 1.8.2, in R to calculate *D*_nn_ and MNTD (Kembel et al., 2010).

### 2.7 Phylogenic community structure

To quantify phylogenetic shape and clustering patterns, we compared our observed phylogenetic structure at the community level to null models of random assemblages from the regional species pool (all species found across all sites) using mean pairwise distance (MPD) and mean nearest taxon distance (MNTD). Where PD_Faith_ measures the sum of all phylogenetic branch lengths at a community, MPD summarizes all phylogenetic distances between species in a tree, capturing deep branching patterns in the phylogeny, whereas MNTD captures patterns in the terminal branches. SES values were again calculated using tip shuffling across the regional phylogeny for 999 iterations. SES values were tested using the p‐value (quantile) of observed vs. null communities [observed rank/(number of runs +1)]. Finally, we examined how varying species richness across our communities impacts evolutionary distances between taxa within the communities. To do this we examined the correlation between species richness and PD_Faith_, MPD, and MNTD using Pearson's correlation coefficient (alpha = 0.05).

### 2.8 Assessing the influence of grazing on diversity metrics

While we were unable to obtain detailed or informative management information for most of our communities, six of them were subjected to grazing, while nine were not (with six communities lacking information) (Table 1). We used both a negative binomial generalized linear model (GLM) and Poisson GLM to explore if grazing presence had an influence on species richness, *D*_nn_ (for all three rarity thresholds), MNTD (across each community and also for rare and common categories defined by all three rarity thresholds), MPD, and PD_Faith_. The dispersion ratio was larger than 1, and the standardized Pearson residuals were smaller for the negative binomial GLM, suggesting the negative binomial GLM model fits better. We report results from the negative binomial GLM below. Goodness‐of‐fit was assessed for each individual negative binomial GLM by calculating Chi‐square (*χ* ^2^; null deviance minus residual deviance). These analyses were conducted using the R package “MASS” version 7.3–56 (Venables et al., 2002).

## 3 RESULTS

**FIGURE 2:**
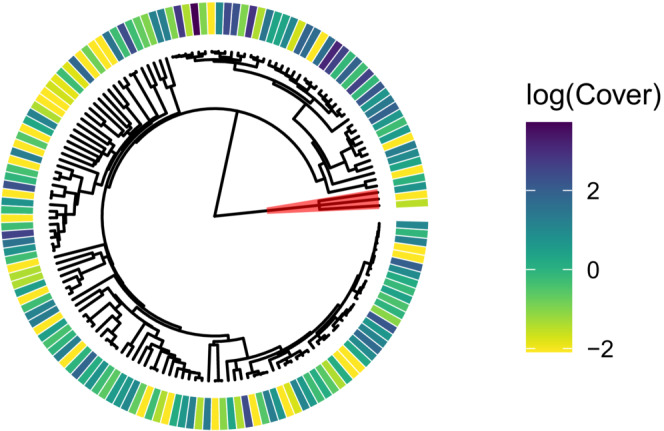
Phylogenetic tree of all species found across all communities. Log‐transformed average percent cover values for each species across each community are denoted on the outer ring with darker colors indicating more abundant species and lighter colors indicating less abundant species. The three *Equisetum* species found on transects are highlighted in red; these species were removed from analyses due to their long branch lengths influencing results.

In total, 928 specimens were collected from the 21 communities representing 47 families and 194 species. Species richness in each community ranged from 9 to 48 (Table 2). The regional phylogenetic tree was created from 152 taxa representing 39 families and 145 species +10 identifications to genus level found in all transects (remaining 40 specimens collected occurred outside of the transect and are not used in analyses; Figure 2). Of the 39 families found in the transects, Asteraceae, Poaceae, and Fabaceae were the most species rich (38, 32, and 14 species, respectively), representing 58% of the total species pool. Of the 10 most abundant genera, 9 were grasses. Only three genera occurred in more than 15% of the area covered by all transects (*Bromus*, *Andropogon*, and *Poa*), while 55% of genera (59 of 107) occurred in less than 1% of the area covered by our transects.

**TABLE 2:** Results for species richness, phylogenetic diversity and structure, breakpoint analysis, and Blomberg's K

Community	# Taxa	Faith's PD		MPD		MNTD		Breakpoints	Blomberg's K
		Observed	SES	Observed	SES	Observed	SES		
7‐mile	32	1866.62	−1.3 (0.107)	231.95	−0.75 (0.348)	73.74	−1.53 (0.067)	15 (0.004**) (−15)	0.07 (0.527)
Altamont Prairie	31	2254.14	−0.15 (0.463)	255.19	0.22 (0.552)	93.41	−0.48 (0.331)	(0.923)	0.17 (0.696)
Brookings Prairie	33	2472.25	0.23 (0.547)	290.59	1.64 (0.912)	85.13	−0.88 (0.205)	4, 11, 15 (<0.001***) (−104)	0.11 (0.169)
Coteau Lake	15	670.98	−2.25 (<0.01**)	180.37	−1.81 (<0.01**)	50.47	−2.51 (<0.01**)	(0.663)	0.31 (0.121)
Coteau Prairie	38	2726.2	0.19 (0.519)	279.8	1.36 (0.858)	85.57	−0.66 (0.272)	5 (0.026*) (−18)	0.04 (0.821)
Cox	15	815.09	−1.75 (<0.01**)	208.88	−1.08 (0.034*)	55.99	−2.35 (<0.01**)	(0.277)	0.43 (0.029*)
Crystal Springs	42	1919.85	−2.65 (<0.01**)	216.74	−1.61 (0.011*)	61.27	−2.35 (0.012*)	26 (0.001***) (−8)	0.18 (0.013*)
Deer Creek	9	698.34	−0.89 (0.116)	217.83	−0.63 (0.162)	102.45	−1.05 (0.145)	(0.87)	0.93 (0.049*)
Gary Gulch E	29	1953.07	−0.67 (0.327)	225.59	−0.87 (0.179)	98.49	−0.26 (0.421)	(0.175)	0.38 (0.022*)
Gary Gulch W	39	2281.99	−1.12 (0.171)	226.04	−1.14 (0.109)	86.74	−0.52 (0.315)	(0.645)	0.2 (0.098)
Hole in the Mountain	25	1279.89	−1.99 (<0.01**)	207.43	−1.52 (<0.01**)	71.57	−1.85 (0.026*)	(0.186)	0.16 (0.584)
Jacobson Fen	38	2900.33	0.65 (0.681)	258.04	0.35 (0.621)	105.03	0.68 (0.731)	27 (<0.001***) (−21)	0.04 (0.798)
Lake Ketchum	19	1010.82	−1.93 (<0.01**)	207.95	−1.27 (0.019*)	67.48	−1.97 (0.016*)	(0.537)	0.25 (0.132)
McKillican	14	923.86	−1.26 (0.046*)	220.55	−0.7 (0.156)	82.06	−1.54 (0.038*)	(0.213)	0.46 (0.131)
Overland	22	1406.45	−1.12 (0.122)	226.35	−0.78 (0.205)	86.91	−1.11 (0.136)	(0.148)	0.32 (0.286)
Punished Woman Grazed	24	1729.97	−0.47 (0.406)	251.08	0.09 (0.629)	83.31	−1.23 (0.118)	(0.098)	0.09 (0.442)
Punished Woman Seeded	23	1199.6	−1.9 (<0.01**)	208.59	−1.37 (0.011*)	65.25	−2.07 (<0.01**)	11 (0.048*) (−1)	0.17 (0.485)
Round/Bullhead	26	1291.2	−2.09 (<0.01**)	213.79	−1.34 (0.018*)	62.91	−2.24 (<0.01**)	(0.702)	0.15 (0.506)
Severson	25	2159.69	0.58 (0.657)	273.1	0.76 (0.707)	110.42	0.08 (0.544)	12 (0.002**) (−12)	0.17 (0.66)
Sioux Prairie	34	1715.18	−1.95 (<0.01**)	217.19	−1.4 (0.024*)	68.62	−1.92 (0.028*)	(0.389)	0.08 (0.924)
Wike	48	3239.31	0.34 (0.568)	246.15	−0.2 (0.368)	93.71	0.48 (0.686)	5, 24, 29 (0.048*) (−43)	0.07 (0.041*)
Regional									0.02 (0.205)

*Note*: *p*‐values are within parentheses, and significance is denoted by asterisks (*p < .05, **p < .01). Breakpoints are listed if applicable, with *p*‐values in parentheses, and ΔBIC values in the second set of parentheses if applicable.

**FIGURE 3:**
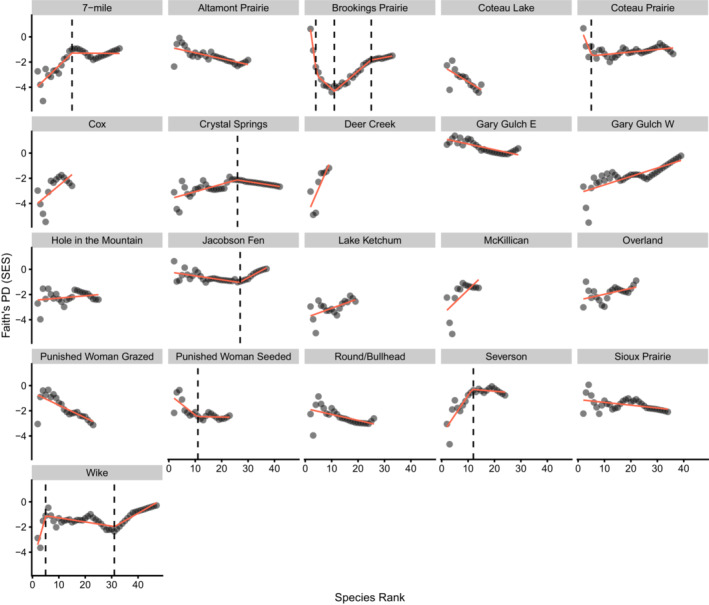
SAPD curves with breakpoint results. Breakpoints are indicated by vertical dashed lines. Red lines are linear models or segmented models depending on breakpoint presence.

When adding increasingly rare species to the phylogenetic tree, we found eight communities with a significant breakpoint (p‐values ≤0.01; Figure 3; Tables 2, S2). The number of breakpoints ranged from one to three, with the location of the breakpoint varying from species rank 4 to rank 31 (Table 2). Three communities (Brookings Prairie, Coteau Prairie, and Wike) had breakpoints early in the SAPD curve (before abundance rank 10), when PD_Faith_ can have drastic changes with the addition of new species. Four communities had positive slopes for their last trendline when the rarest species are added (Brookings Prairie, Coteau Prairie, Jacobson Fen, and Wike), however, Brookings Prairie and Coteau Prairie were weak (slope was between 0 and 0.1). The remaining four communities had weak, negative trends for their last trendline (slope was between −0.1 and 0).

The phylogenetic signal of abundance categories using Blomberg's K statistic was extremely low and insignificant when looking at all species in the regional species pool at 0.02 (*p*‐value = .21), indistinguishable from a random expectation. When calculating Blomberg's K statistic by the community, we found all K < 1, ranging from 0.04 (Coteau Prairie) to 0.93 (Deer Creek) (Table 2). Only five communities showed significant phylogenetic signal (Cox, *p*‐value = .031; Crystal springs, 0.013; Deer Creek, 0.049; Gary Gulch E, 0.013; and Wike, 0.044), with K ranging from 0.07 to 0.93 across these communities.

**FIGURE 4:**
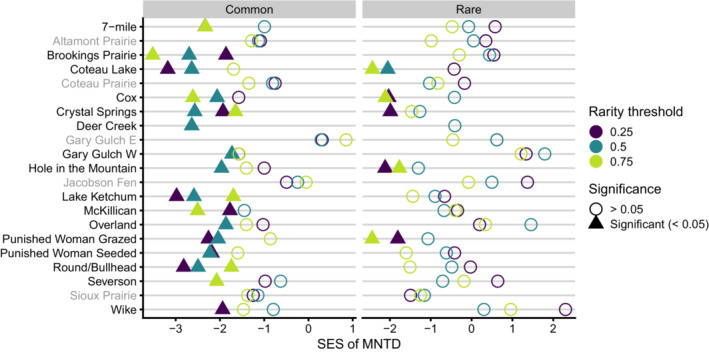
Standardized effect sizes (SES) of MNTD for rare and common species across each community and three rarity threshold values. The color indicates rarity threshold, while the shape indicates significance (triangle = significant). Communities with no significant SES values are gray.

**FIGURE 5:**
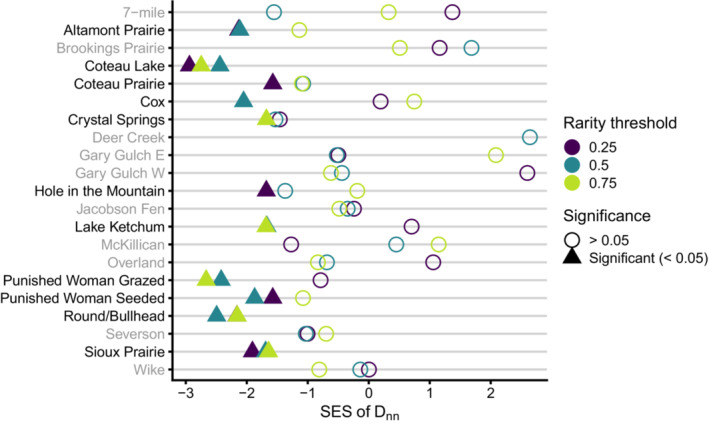
Standardized effect sizes (SES) of *D*_nn_ across each community and three rarity threshold values. The color indicates the rarity threshold, while the shape indicates significance (triangle = significant). Communities with no significant SES values are gray.

When using the most restrictive rarity classification of 0.25, we found 10 communities with significant phylogenetic clustering of common species (7‐mile, *p*‐value = .007; Brookings Prairie, 0.033; Coteau Lake, 0.002; Crystal Springs, 0.029; Lake Ketchum, 0.002; McKillican, 0.036; Punished Woman Grazed, 0.013; Punished Woman Seeded, 0.013; Round/Bullhead, 0.006; and Wike, 0.03), and four communities with significant phylogenetic clustering of rare species (Cox, *p*‐value = .031; Crystal Springs, 0.014; Hole in the Mountain, 0.019; and Punished Woman Grazed, 0.044; Figure 4; Table S3). Using the moderate rarity classification of 0.5 resulted in 12 communities with significant clustering of common species (Brookings Prairie, *p*‐value = .003; Coteau Lake, 0.005; Cox, 0.019; Crystal Springs, 0.003; Deer Creek, 0.007; Gary Gulch W, 0.047; Hole in the Mountain, 0.021; Lake Ketchum, 0.005; Overland, 0.023; Punished Woman Grazed, 0.022; Punished Woman Seeded, 0.009; and Round/Bullhead, 0.004), and one community with significant clustering of rare species (Coteau Lake, *p*‐value = .02). Finally, using a rarity classification of 0.75 resulted in seven communities with significant clustering of common species (7‐mile, *p*‐value = .006; Brookings Prairie, 0.001; Cox, 0.0075; Crystal Springs, 0.048; McKillican, 0.011; Round/Bullhead, 0.045; and Severson, 0.016) and four communities with significant clustering of rare species (Coteau Lake, *p*‐value = .006; Cox, 0.01; Hole in the Mountain, 0.044; and Punished Woman Grazed, 0.006). We found common and rare species were significantly more closely related to each other than expected from random at seven communities when using a rarity classification of 0.25 (Altamont Prairie, *p*‐value = .01; Coteau Lake, 0.001; Coteau Prairie, 0.039; Hole in the Mountain, 0.032; Punished Woman Seeded, 0.041; Round/Bullhead, 0.007; and Sioux Prairie, 0.01), eight when using 0.5 (Altamont Prairie, *p*‐value = .013; Coteau Lake, 0.003; Cox, 0.014; Lake Ketchum, 0.037; Punished Woman Grazed, 0.002; Punished Woman Seeded, 0.023; Round/Bullhead, 0.004; and Sioux Prairie, 0.035), and six when using 0.75 (Coteau Lake, *p*‐value = .003; Crystal Springs, 0.026; Lake Ketchum, 0.028; Punished Woman Grazed, 0.001; Round/Bullhead, 0.004; and Sioux Prairie, 0.023; Figure 5; Table S3). Only one community (Deer Creek) using rarity thresholds of 0.25 and 0.75 was removed due to an insufficient number of species in rare and common categories (<4). Overall, these results indicate rare species are closely related to each other, as are common species to each other and too rare species.

**FIGURE 6:**
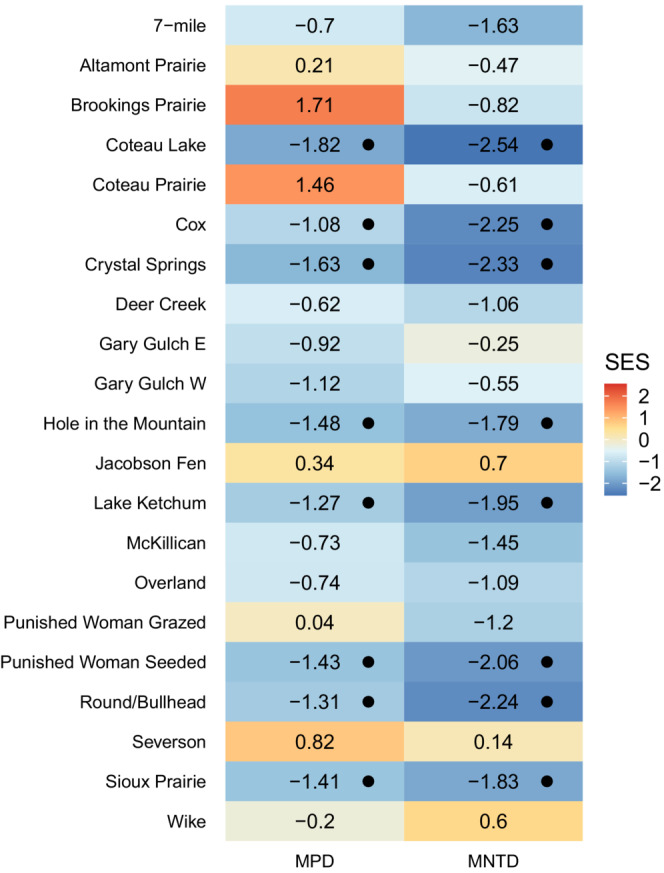
Standardized effect sizes for MPD and MNTD for each community. Color indicates size of SES value (warmer = positive and cooler = negative). Black points indicate significant values.

**FIGURE 7:**
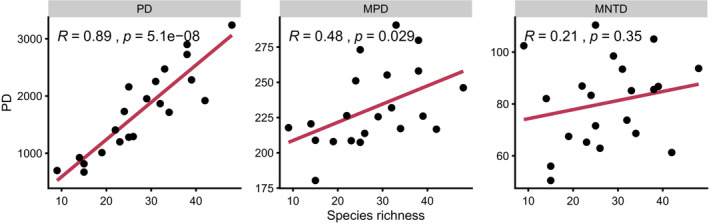
Correlation between species richness and PD_Faith_, MPD, and MNTD.

Phylogenetic diversity was generally lower than expected when compared to the regional phylogeny (phylogenetic clustering) with eight and nine communities exhibiting significant phylogenetic clustering when using SES_MPD_ and SES_MNTD_, respectively (MPD: Coteau Lake, p‐value = 0.001; Cox, 0.034; Crystal Springs, 0.011; Hole in the Mountain, 0.004; Lake Ketchum, 0.019; Punished Woman Seeded, 0.011; Round/Bullhead, 0.018; Sioux Prairie, 0.024; MNTD: Coteau Lake, 0.001; Cox, 0.003; Crystal Springs, 0.012; Hole in the Mountain, 0.026; Lake Ketchum, 0.016; McKillican, 0.038; Punished Woman Seeded, 0.009; Round/Bullhead, 0.009; and Sioux Prairie, 0.028; Table 2; Figure 6). While some communities had positive SES values (phylogenetic overdispersion), none were significant. Communities that had significant phylogenetic clustering for rare and common species separately were generally the same communities that had phylogenetic clustering for all species (Figures 4, 5, 6). We found strong correlation between PD_Faith_ and species richness (*R* = 0.89, *p*‐value = <.001), no correlation between MNTD and species richness (*R* = 0.21, *p* = .35), and weak but significant correlation between MPD and species richness (*R* = 0.48, *p* = .029; Figure 7).

Using a negative binomial GLM, we were unable to detect an influence of grazing on all tested metrics (species richness, PD_Faith_, MPD, MNTD, D for the three rarity thresholds, and MNTD for both rare and common categories under all three rarity thresholds). The *p*‐values for each individual negative binomial GLM were not significant and well above 0.1 (Table S4).

## 4 DISCUSSION

Patterns involving the assembly and maintenance of species in a community remain an open question in ecology. Rare species are among the most vulnerable to local extirpation (Purvis et al., 2000), and investigations into how they shape phylogenetic and functional diversity allow us to understand the consequences of their loss from the community and inform management strategies moving forward. Focusing on 21 tallgrass prairie communities across the Prairie Coteau in the northern Great Plains, we found overall that rare species do not contribute significantly to phylogenetic diversity but may provide redundancy with more common species and with other rare species. Thus, patterns in phylogenetic diversity across these prairie communities do not support the niche differentiation hypothesis for the coexistence of rare and common species (Gaston, 1994; Grime, 1998; Hanski, 1982).

Niche differentiation predicts that species coexistence is maintained by resource partitioning and functional differences (e.g., Hubbell (2005). However, only eight of our communities had significant breakpoints detected and of those, only three of those had an increasing PD_Faith_ when the rarest species were added. Trendlines in the remaining communities were variable, with six of these communities without breakpoints having decreasing PD_Faith_ as rarer species were added, indicating they were more closely related to common species than expected from random. When examining Blomberg's K statistic, we did not find evidence for a phylogenetic signal of abundance categories across our regional phylogeny, although five of our communities did show significant phylogenetic signal with a *K* < 1, indicating non‐random evolution of abundance. These results also correspond to our results for *D*_nn_ and MNTD, which indicate close relatedness between and within rare and common species.

Although “phylogeny‐as‐proxy” assumptions for ecological/functional similarity have been questioned (e.g., Gerhold et al., 2015), evolutionary relatedness can provide initial insight into the broad patterns in community structure, as we have done here. This can be especially useful when trait data are lacking, as is common for rare species (Swenson, 2013). Moreover, phylogenetic diversity and functional diversity are often highly positively correlated (Cadotte et al., 2019), and a strong connection between these metrics was found in tallgrass prairies (Larkin et al., 2015). Although we did not directly test the connection between phylogenetic diversity and functional traits, our results of phylogenetic redundancies between species echo previous work on functional redundancies in tallgrass prairies of the Great Plains (Jain et al., 2014). Redundancy in grasslands could be an essential mechanism for ecosystem stability, where a range of functionally redundant rare species differing slightly in their ability to respond to disturbances, invasion, and climatic changes can persist on the landscape (MacDougall et al., 2013). Findings from Jain et al. (2014) and MacDougall et al. (2013) indicate possible increases in functional contributions of rare species if they become more abundant under a changing climate and increasingly disturbed landscape.

Communities can become phylogenetically clustered due to environmental filtering from high disturbance levels, whether natural or human caused, such as plowing, grazing, or fire (Brunbjerg et al., 2012; Dinnage, 2009; Grime, 1973; Webb, 2000) or through recent species radiations (Le Bagousse‐Pinguet et al., 2019). Due to limited water, reliance on disturbance, and seasonality, temperate prairies are likely historically phylogenetically clustered (Kerkhoff et al., 2014; Massante et al., 2019; Wiens & Donoghue, 2004) and, with increased anthropogenic disturbance and climate change, are likely to become increasingly less phylogenetically diverse (Larkin et al., 2015; Li et al., 2019a; Zhu et al., 2019). This suggests disturbance regimes may play a role in how redundant (or not) rare species are with common species. We found significant phylogenetic clustering using both MPD and MNTD at 8 and 9 of 21 communities for MPD and MNTD, respectively, indicating species across ~40% of the communities were more closely related to each other than expected from a random sampling of the regional phylogeny.

Our finding of phylogenetic clustering is unsurprising, as most species, both common and rare, were those occurring in the species‐rich clades that tend to dominate grassland communities (i.e., Poaceae, Asteraceae, and Fabaceae). Species within these clades also demonstrate phylogenetic clustering in tallgrass prairies (Kellar et al., 2015). Of the forest plots analyzed by Mi et al. (2012), those dominated by disturbance all rejected the niche differentiation hypothesis by showing rare species were not phylogenetically distinct from common species or each other. When we tested for the influence of one management type, grazing presence, on several phylogenetic diversity metrics (PD_Faith_, MNTD, MPD, and *D*_nn_), we found no support for this relationship. However, our sampling was limited to the tracts of land we had permission to survey and included differences in management (e.g., grazing, burning, and historical tilling), with little opportunity for replication in our experimental design. Moving forward, careful replication of historic and current management types will be needed to fully understand the interactions between disturbance regimes and the coexistence of rare and common species.

Previous work has found a correlation between phylogenetic diversity metrics (predominantly PD_Faith_) and species richness, indicating species richness can act as a proxy for phylogenetic diversity in conservation prioritization (Brooks et al., 2006; Kellar et al., 2015; Rodrigues et al., 2005; Tucker & Cadotte, 2013), however, other work has indicated weak correlations (Brum et al., 2017; Daru et al., 2019; Forest et al., 2007; Pollock et al., 2017). Possible discrepancies in the correlation between phylogenetic diversity and species richness have been linked to the topology of the regional phylogenetic tree, where ancient speciation events (long terminal branches) and evenly distributed branch lengths across clades result in stronger correlations between phylogenetic diversity and species richness (Cadotte et al., 2010; Rodrigues et al., 2005; Tucker & Cadotte, 2013). We found a strong correlation between PD_Faith_ and species richness, despite evidence of phylogenetic clustering. However, we do not find this relationship with phylogenetic structure metrics. We found weak to no correlation between species richness and community MNTD and MPD, indicating species‐rich communities may not have increased diversity at the terminal branches of the phylogeny (MNTD) and only marginal improvements in diversity at the deeper branches of the tree (MPD). Our results indicate caution is needed when using species richness as a surrogate for phylogenetic diversity in tallgrass prairie communities, especially when considering the terminal or basal branches of the phylogenetic tree.

With most species in our communities occurring in three main families (Poaceae, Asteraceae, and Fabaceae), adding species outside of these clades is more likely to significantly increase phylogenetic diversity. The 30 longest terminal branches of our regional tree were species outside Asteraceae, Poaceae, and Fabaceae, possibly indicating utility for restoration managers to consider adding species outside of these three families to increase phylogenetic diversity, as has been suggested by Barak et al. (2017), Cavender‐Bares and Cavender (2011), and Hipp et al. (2015). Evidence indicates current restoration practices result in lower phylogenetic diversity than untilled, native remnants, and are not functionally equivalent (Barak et al., 2017). Including phylogenetic diversity in management and restoration practices could promote system resiliency and lead to higher success rates of restoration and management (Barber et al., 2019; Hipp et al., 2015; Karimi et al., 2021), an especially important consideration in the face of an ongoing extinction event and changing climate.

Sampling techniques are an important consideration in contextualizing our results. This study was conducted as a snapshot of the Prairie Coteau's tallgrass prairies in August 2019, capturing predominantly warm‐season species. Additionally, 2019 experienced one of the wettest years on record for the region (NWS Sioux Falls NOAA), which may influence species' presence and abundance. A sampling at one time of the growing season has the potential to influence observed phylogenetic diversity by under‐sampling taxonomic diversity (Jantzen et al., 2019; Park et al., 2018) due to temporal phenological niche separation and phenological conservatism (i.e., flowering time) (Kochmer & Handel, 1986; Wright & Calderon, 1995). However, our sampling methods included all living individuals, regardless of phenology, reducing potential sampling effects. The effect of seasonally biased sampling on phylogenetic diversity metrics was outside the scope of this project but would be beneficial for future studies to optimize sampling techniques.

Our findings indicate tallgrass prairies may be resilient to ongoing climatic changes and human disturbance due to functional redundancy, but only so far as there are species to fulfill this redundancy. Conservation of species, both rare and common, is essential to protecting ecosystem functionality and viability for the long term. We are currently experiencing unprecedented species loss, particularly in grassland communities (Hoekstra et al., 2005), that could lead to catastrophic ecological decline if we do not act to conserve extant species. Rare species are understudied, with numerous species having very little data (Whittaker et al., 2005), severely impeding our scientific understanding and management ability. Phylogenetic diversity could serve as a helpful tool to estimate functional traits and diversity in communities, especially for rare species that are less likely to have functional trait data (Jain et al., 2014). While our results do not indicate rare species contribute high levels of evolutionary history to communities, it is imperative that we continue to account for less common species for the long‐term health and survival of the critically threatened tallgrass prairies of North America.

## AUTHOR CONTRIBUTIONS

**Sarah Herzog:** Conceptualization (supporting); data curation (lead); formal analysis (lead); funding acquisition (supporting); investigation (lead); methodology (equal); project administration (equal); visualization (lead); writing – original draft (lead); writing – review and editing (equal). **Maribeth Latvis:** Conceptualization (lead); data curation (supporting); formal analysis (supporting); funding acquisition (lead); investigation (supporting); methodology (equal); project administration (equal); writing – original draft (supporting); writing – review and editing (equal).

## CONFLICT OF INTEREST

None declared.

## Supporting information


Table S1

Table S2

Table S3

Table S4

## Data Availability

Data and code are available in Dryad (https://doi.org/10.5061/dryad.x0k6djhgz).
